# Value-Based Contracting in Clinical Care

**DOI:** 10.1001/jamahealthforum.2024.2020

**Published:** 2024-08-23

**Authors:** Claire Boone, Anna Zink, Bill J. Wright, Ari Robicsek

**Affiliations:** 1 Booth School of Business, University of Chicago, Chicago, Illinois; 2 Providence Research Network, Portland, Oregon

## Abstract

This cohort study analyzes quality incentives, requirements, and other measures in employment and payer contracts of primary care clinicians.

## Introduction

Value-based contracts are popular for quality improvement in primary care despite mixed evidence of their effectiveness.^1,2,3^ Explanations for their underperformance include complexity of health care, misalignment of measures, and inadequate financial incentives.^1,2,3^ Another potential unexplored factor is volume of quality measures, especially if clinicians face multiple contracts featuring different quality measures and reporting requirements. We quantified the number and diversity of quality measures and value-based contracts faced by primary care physicians (PCPs).

## Methods

We obtained employment contract data on PCPs continuously employed by an integrated health system from 2020 to 2022 along with payer contracts associated with their attributed patients. Patients who interacted with the health system in the previous 2 years were attributed to 1 PCP annually and linked to a payer contract based on their insurance plan at year end (eMethods in Supplement 1). The Providence Research Network Institutional Review Board approved the study and waived informed consent because it was not considered human participant research. We followed the STROBE reporting guidelines.

Payer contract data included type (commercial, Medicaid, or Medicare), incentivized process- or outcome-based quality measures, and Health Care Payment Learning & Action Network (HCPLAN) category.^4^ HCPLAN categories 2C, 3A, and 3B were considered value-based contracts.

We measured the number of unique value-based contracts and quality measures per physician-year based on their assigned patients. Quality measures were considered distinct if they referenced different conditions, and measures for the same condition were considered distinct if the value differed (eg, hemoglobin A_1c_ <8% vs <9% [to convert to proportion of total hemoglobin, multiply by 0.01]). We conducted 2-sample *t* tests to assess changes in exposure to value-based contracts and quality measures across years; *P* < .05 indicated statistical significance. Results were robust to excluding PCPs with small panels.

Quality measure information was missing for 29% of value-based contracts with attributed patients; thus, we reported both mean number of contracts and mean number of contracts with nonmissing quality measures per physician-year. Analyses were run with R 4.4.0 (R Core Team).

## Results

The 809 PCPs included (519 females [58.3%], 371 males [41.7%]) had a mean (SD) of 1308.71 (622.73) attributed patients and an increasing number of value-based contracts from 2020 to 2022 (9.39 to 12.26; *P* < .001) (Table). Contracts contained a mean (SD) of 10.24 (2.66) quality measures. Physicians faced a mean (SD) 57.08 (24.58) unique quality measures across 7.62 (5.08) value-based contracts (Table). Distinct measures per physician ranged from 0 to 103 (Figure). Medicare contracts had more quality measures on average than commercial or Medicaid contracts (13.42 vs 10.07 or 5..37). The mean (SD) number of quality measures in Medicare contracts increased significantly from 13.14 (4.72) in 2020 to 15.04 (3.99) in 2022 (*P* < .001) (Table).

**Table.** Changes in Value-Based Care Contracts and Quality Measures Over Time^a^

	Mean (SD)				Difference between 2022 and 2020	*P* value^b^
	Overall	2020	2021	2022		
PCPs, No.^c^	890	890	890	890	NA	NA
No. of patients in physician’s panel	1308.71 (622.73)	1272.17 (601.76)	1336.39 (606.09)	1317.58 (657.73)	45.42	.13
Among contracts in a physician’s patient panel						
No. of value-based contracts	11.18 (5.70)	9.39 (5.28)	11.89 (5.43)	12.26 (5.94)	2.87	<.001
No. of value-based contracts with nonmissing quality measure data	7.62 (5.08)	7.79 (5.71)	8.31 (4.66)	6.74 (4.67)	−1.05	<.001
No. of unique quality measures	57.08 (24.58)	54.78 (24.75)	64.08 (24.71)	52.37 (22.67)	−2.41	.03
Among value-based contracts in a physician’s patient panel						
Share of contracts by payer type, %^d^						
Commercial	49.50	55.02	48.64	44.65	−0.10	<.001
Medicaid	21.49	14.78	17.68	32.43	0.18	<.001
Medicare	29.01	30.20	33.68	22.93	−0.07	<.001
No. of measures per contract^e^						
Overall	10.24 (2.66)	10.23 (2.14)	10.38 (2.92)	10.11 (2.85)	−0.12	.31
Commercial	10.07 (2.29)	9.92 (1.50)	10.69 (2.43)	9.58 (2.66)	−0.34	.002
Medicaid	5.37 (1.00)	4.54 (0.88)	5.61 (0.53)	5.70 (1.10)	1.16	<.001
Medicare	13.42 (4.72)	13.14 (4.72)	12.42 (4.92)	15.04 (3.99)	1.91	<.001

Abbreviations: NA, not applicable; PCP, primary care physician.

^a^ Value-based contracts were Health Care Payment Learning & Action Network categories 2C, 3A, and 3B.

^b^ *P* value from a 2-sided *t* test.

^c^ The PCPs were employed by the health system for all 3 study years.

^d^ Denotes statistics among all value-based contracts.

^e^ Denotes statistics among value-based contracts with nonmissing quality metrics data.

**Figure.**
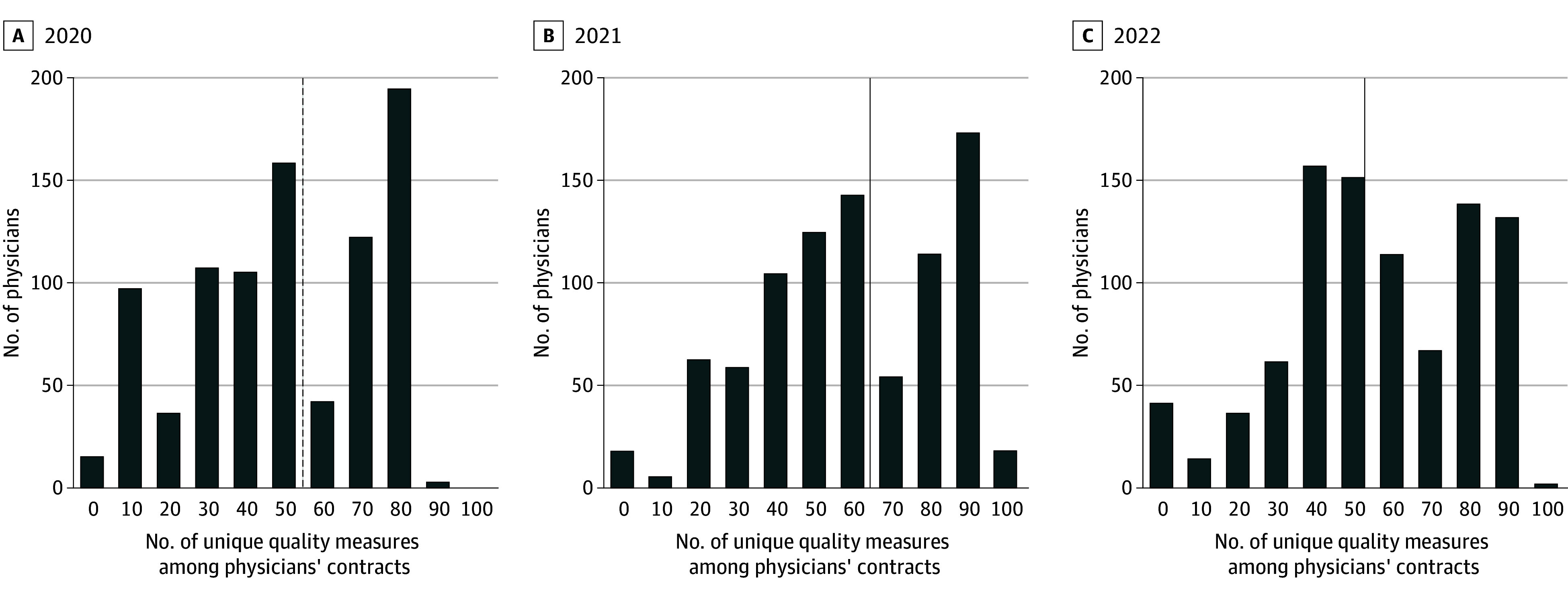
Number of Unique Quality Measures Among Physicians' Primary Care Patient Contracts, by Year Sum of unique quality measure among value-based care contracts in primary care physicians’ patient panels (n = 890 physicians). A unique quality measure is, for example, share of patients with blood pressure under 140/90 mm Hg. If the same metric appeared in multiple contracts, it was counted once. Vertical lines represent the mean number of quality measures per physician: 54.8, 64.1, and 52.4 in 2020, 2021, and 2022 respectively.

## Discussion

Previous research on value-based contracts suggests these models have not lived up to their potential.^1,2,3^ We found saturation of the quality measure environment as a possible explanation: average physicians were incentivized to meet 57.08 different quality measures annually.

Study limitations include estimates that were likely lower bounds on PCPs’ exposure to quality measures. Physicians often face additional quality measures in their employment contracts, but our data on contracts’ quality measures were incomplete. Percentage of missing data was lower in 2021, which may explain the larger number of unique quality measures that year. Additionally, the data source was an integrated health system with multiple payers; thus, findings may not generalize to other settings.

Value-based contracting is intended to incentivize care improvement, but it is unlikely a clinician or practice can reasonably optimize against 50 or more measures at a time. Increased use of such levers may also carry unintended consequences. Clarity and salience are crucial to changing behavior,^5^ and the burden of extraneous information and processes has been increasingly associated with adverse outcomes, such as physician burnout.^6^ As payers increasingly shift toward value-based contracts, additional research is needed to understand how their ubiquity affects their benefits and how such contracts can be scaled sustainably for clinical care.

## Notes

Supplement 1. **eMethods.** Sample and Attribution of Patients to Physicians

Supplement 2.
Data Sharing Statement
