# Supplementary material for: Value-Based Contracting in Clinical Care
Source: JAMA Health Forum. 2024 Aug 23;5(8):e242020. doi: 10.1001/jamahealthforum.2024.2020 (PMC11344232; doi:10.1001/jamahealthforum.2024.2020)
Supplement: Supplement 1. — eMethods. Sample and Attribution of Patients to Physicians [file jamahealthforum-e242020-s001.pdf]

Supplemental Online Content

Boone C, Zink A, Wright BJ, Robicsek A. Value-based contracting in clinical care. *JAMA Health Forum.* 2024;5(8):e242020. doi:10.1001/jamahealthforum.2024.2020

eMethods. Sample and Attribution of Patients to Physicians

This supplemental material has been provided by the authors to give readers additional information about their work.

© 2024 Boone C et al. *JAMA Health Forum.*

eMethods. Sample and Attribution of Patients to Physicians

We started with all primary care providers who were continuously employed by the health system from 2020 through 2022. After excluding non-physicians, and urgent care physicians, only primary care physicians were included in our final sample. This includes primary care physicians who were primary care only as well as primary care physicians with specialty status, which could include internal medicine, family medicine, or pediatrics. Providers who had been employed less than 24 months were excluded, and providers whose panels sum to 100 patients or less are excluded from patient panel data in the health system's records.

We used the health system's logic for attributing a patient to a primary care physician panel, which was based off the Epic field "Primary Care Provider". A paneled patient is defined as one who is alive, with a primary care provider assigned, and had one or more outpatient service in the past two years.

© 2024 Boone C et al. *JAMA Health Forum.*
