# Supplementary material for: Value-Based Contracting in Clinical Care
Source: JAMA Health Forum. 2024 Aug 23;5(8):e242020. doi: 10.1001/jamahealthforum.2024.2020 (PMC11344232; doi:10.1001/jamahealthforum.2024.2020)
Supplement: Supplement 2. — Data Sharing Statement [file jamahealthforum-e242020-s002.pdf]

Data Sharing Statement

Boone. Value-Based Contracting in Clinical Care. *JAMA Health Forum*. Published August 23, 2024. doi:10.1001/jamahealthforum.2024.2020

Data
Data available: No
